# Global trends in osteoimmunology and osteoporosis research: A bibliometric analysis from 2013 to 2022

**DOI:** 10.1097/MD.0000000000042367

**Published:** 2025-05-02

**Authors:** Bencheng Yang, Mingshuai Tan, Fusheng Xiong

**Affiliations:** a School of Clinical Medicine, Chengdu University of Traditional Chinese Medicine, Chengdu, Sichuan, China; b Department of Spine Surgery, Suining Municipal Hospital of Traditional Chinese Medicine, Suining, Sichuan, China.

**Keywords:** bibliometrics, CiteSpace, osteoimmunology, osteoporosis, Vosviewer

## Abstract

**Background:**

A large number of studies have shown that osteoporosis is closely related to bone immunology. The purpose of this study is to conduct bibliometrics and visual analysis of the fields related to osteoimmunology and osteoporosis from 2013 to 2022 and to summarize the research hotspots and trends in this field.

**Methods:**

We searched the Web of Science core collection database for articles on osteoimmunology and osteoporosis published between 2013 and 2022. Vosviewer 1.6.18 and CiteSpace.6.2. R4 were used to analyze the retrieved data.

**Results:**

A total of 3218 articles on osteoimmunology and osteoporosis were included in this study. A total of 76 countries, 347 institutions, and 502 authors were included in the articles examined in this study. The main research countries were China, the United States, and South Korea. Shanghai Jiaotong University, Harvard University, and the University of California system were the main research institutions. The author who published the most papers was Xu, Jiake.

**Conclusions:**

This study is the first to summarize the global research trends in the field of osteoimmunology and osteoporosis from 2013 to 2022. That helps researchers quickly understand the research hotspots and directions in this field.

## 1. Introduction

Osteoporosis (OP) is a systemic bone disease characterized by reduced bone mass, damage to bone tissue microstructure, increased bone fragility, and susceptibility to fracture.^[1]^ With the aging of the global population, the current 200 million patients with OP will increase each year.^[2]^ In addition, there are more than 9 million cases of osteoporotic fractures worldwide each year, and the number of such patients will double by 2040.^[3]^ In the United States, there are 53.6 million people over the age of 50 with OP, accounting for approximately 54% of the total population in this age group.^[4]^ According to statistics, the annual cost of treating OP in the United States has reached $25.3 billion.^[5]^ The treatment of OP is divided into nondrug measures and drug treatment. Nondrug interventions include avoiding smoking and excessive drinking, weight-bearing and resistance exercises to promote bone formation, and balance training to prevent falls.^[6]^ Drug therapies include vitamin D, calcium, bisphosphonates, RANK ligand inhibitors, parathyroid analogs, estrogen receptor agonists and antagonists, estrogen, and calcitonin.^[7]^

Osteoporosis includes primary osteoporosis and secondary osteoporosis. The possible causes include heredity, immunity, environment, endocrine, medicine, age, and sex.^[8,9]^ According to the previous pathophysiological mechanism, the endocrine mechanism plays a leading role in the occurrence of osteoporosis. For example, changes in dietary structure, vitamin D deficiency, and estrogen deficiency are the key factors for postmenopausal osteoporosis.^[10]^ In female patients, the rapid decline in menopausal estrogen levels is the main cause of primary postmenopausal primary osteoporosis. In the early menopause, due to the decrease in the number of osteocyte differentiation and the increase in apoptosis, cortical bone, and trabecular bone are rapidly lost. In the middle and late stages of menopause, the decreased activity of bone cells leads to their long-term chronic loss.^[11]^ There are many pathological factors of secondary osteoporosis, including diabetes, inflammatory bowel disease, arthritis, parathyroid disease, and the use of glucocorticoids.^[12]^ At present, people realize that the influencing factors of osteoporosis are not limited to this. Intestinal microbiota also plays an important role in the occurrence of osteoporosis. It can affect the production and absorption of human nutrients, the growth of the host, regulate the immune homeostasis of the host, and play an important role in regulating the homeostasis of bone metabolism.^[12–14]^ The pathological mechanism of OP is abnormal osteoclast (OC) differentiation, bone resorption is greater than bone formation, and bone remodeling is unbalanced.^[15]^ OCs are mainly differentiated from monocyte/macrophage precursor cells.^[16]^ The key signaling pathway of OC differentiation is nuclear factor-kappa-B ligand (RANKL)/RANK/osteoprotegerin (OPG), and nuclear factor-κB ligand (RANKL) and macrophage colony stimulating factor (M-CSF) are the key regulatory molecules.^[17–19]^ In 2000, Arron proposed the concept of osteoimmunology. Numerous studies have shown that there are many regulatory molecules between the bone and the immune system.^[20]^ At present, in studies on osteoimmunology, some scholars have pointed out that the immune system plays an important role in the formation mechanism of OP. The immune system and immune cytokines jointly regulate the occurrence and development of osteoporosis.^[21,22]^ The microenvironment of bone cells can provide favorable conditions for the development of the immune system, which can regulate bone metabolism through B cells, T cells, and dendritic cells.^[21,23,24]^ Abnormal activation of the immune system breaks the balance of differentiation between osteoblasts and osteoclasts, leading to abnormal bone remodeling and osteoporosis.^[20,25]^ In addition, the immune system also regulates bone metabolism through inflammatory cytokines and related ligands, thereby affecting bone formation and bone resorption.^[26]^ Among them, the RANKL–RANK–OPG pathway plays a key role in bone and immunity, which plays an important role in the emergence of the field of bone immunology.^[27]^ T cells, macrophages, B cells, and other immune cells, as well as a large number of immune factors, are involved in the process of bone remodeling.^[21–23]^ And studies have shown that T lymphocytes play a key regulatory role in osteoporosis.^[25]^ Moreover, studies have shown that T lymphocytes play a regulatory role in OP.^[28]^ Activated T cells can secrete a variety of cytokines, such as IL-6, IL-7, IL-8, and TNF-α, and these cytokines play a crucial role in the regulation of OC differentiation.^[22,29]^ Animal experiments have found that T cell deficiency in nude mice showed enhanced osteoclast differentiation and accelerated bone loss, which may be due to the immune imbalance of T cells to promote osteoclast differentiation and bone resorption.^[30]^ Th17 cells are mainly involved in promoting the formation of osteoclasts, while Treg cells are mainly involved in inhibiting the differentiation of monocytes into osteoclasts. The imbalance of Th17-Treg cells is one of the important causes of osteoporosis.^[31–33]^ Therefore, immune imbalance promotes osteoclast formation and accelerates bone loss, but the regulatory mechanism of T cells in osteoporosis and the bone system needs more basic experimental studies to prove. At present, the study of T cell therapy in the treatment of osteoporosis has aroused the interest of scholars.^[34]^ T cell therapy may be an important method for the treatment of osteoporosis in the future, which has great potential for development.

With increasing population aging and the introduction of the concept of osteoimmunology, the relationship between osteoimmunology and OP has attracted the attention of scholars. In previous bibliometric studies, Wan et al conducted a bibliometric review of the relationship between osteoarthritis and osteoporosis, pointing out that the main research trends in the future are inflammatory factors, mesenchymal stem cells, and gene expression.^[35]^ Li et al conducted a bibliometric and visual analysis of exercise and osteoporosis and pointed out that “skeletal muscle,” “sarcopenia,” and “mesenchymal stem cells” are future research directions.^[36]^ Jiang et al conducted a visual analysis of the literature on glucocorticoid-induced osteoporosis in the past decade, pointing out that the current hot spots and trends in this field are the mechanism of mesenchymal stem cells in the treatment of the disease.^[37]^ At present, there is still a lack of research on bibliometrics and visual analysis in the field of osteoimmunology and osteoporosis. Bibliometrics is a quantitative analysis of knowledge in a certain field using statistical and mathematical methods.^[38]^ These data can be better understood through bibliometric mapping and visual analysis.^[39]^ The purpose of this study is to systematically review the research trends and hotspots of osteoimmunology and OP between 2013 and 2022 and to fill the gaps in this field.

## 2. Methods

### 2.1. Data source

**Figure 1.**
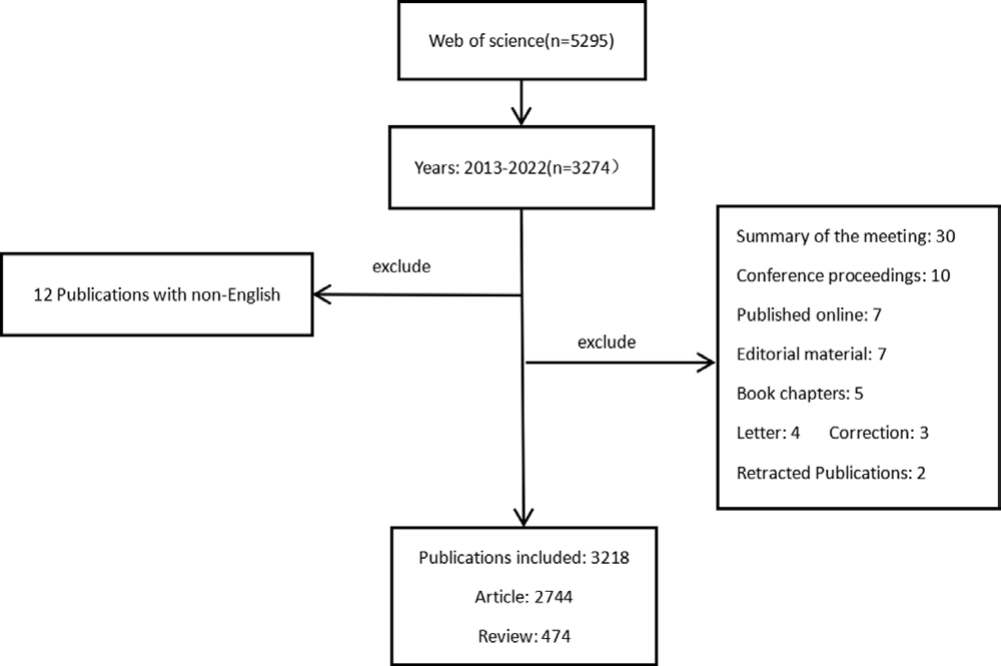
Flow diagram of the included articles.

Data were retrieved from the Web of Science Core Collection (WoSCC) because it can provide more comprehensive information than other sources.^[40]^ The search period was set to “January 01, 2013” to “December 31, 2022.” The type of retrieved literature was set to “article or review.” The search language was set to “English.” The retrieval process was completed on September 3, 2023, to prevent data updates from causing errors. The search formula was as follows: TS = (osteoimmunology OR macrophage OR neutrophil OR “NK cell” OR “natural killer cell” OR “dendritic cell” OR DC OR “innatelymphoid cells” OR ILCs OR “T cell” OR “T lymphocyte” OR “B cell” OR “B lymphocyte” OR “regulatory T cell” OR “Treg” OR monocyte OR “immune dysfunction” OR “immune response”) AND TS = (osteoporosis OR osteopenia OR osteoporoses OR “bone loss*” OR “low bone density” OR “low bone mass”). Finally, the retrieved records are exported in plain text format for further bibliometric research. The retrieval process was completed independently by 2 researchers. The detailed retrieval steps are shown in Figure 1.

### 2.2. Data analysis

This study used Vosviewer 1.6.18 and CiteSpace.6.2. R4 and Microsoft Office Excel 2010 to analyze the data. Vosviewer 1.6.18 was used to analyze journals, cocited references, and highly cited references. CiteSpace.6.2. R4 was used to analyze the number of authors, institutions, countries or regions, keywords, and publications. The parameter settings and results of CiteSpace are the same as previous studies.^[41]^ The specific parameter settings of CiteSpace.6.2. R4 are as follows: the time period is set from the first day of 2013 to the last day of 2022; the term source is set as title, abstract, author keywords, and keyword plus; the node type is set as author, institution, country or region, keywords, references, cited author, and journal; and the link strength is set as cosine. In addition, Microsoft Office Excel 2010 is used for data collation and table production. In addition, Microsoft Office Excel 2010 was used for data collation and table production.

## 3. Results

### 3.1. Publication and trend analysis

**Figure 2.**
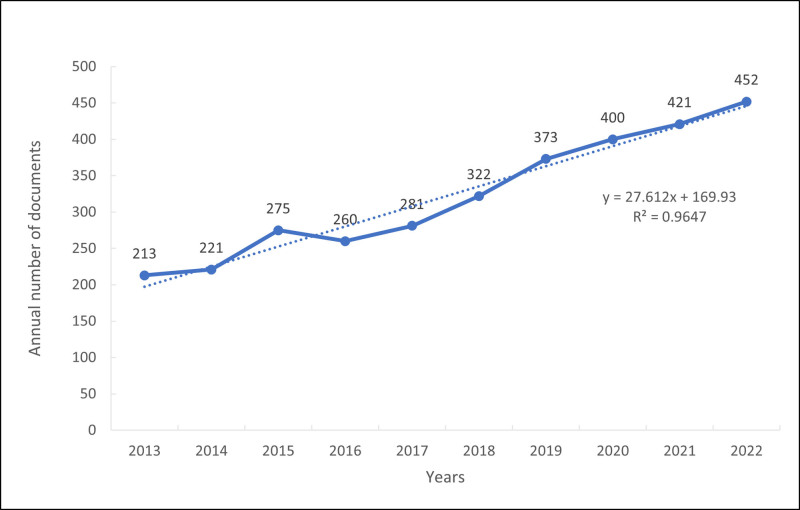
Published trend chart concerning osteoimmunology and osteoporosis.

A total of 3218 papers were included in this study, including 2744 articles and 474 reviews. As shown in Figure 2, from 2013 to 2022, the number of articles published on osteoimmunology and OP continued to grow steadily. This suggests that an increasing number of scholars began paying attention to this field. Moreover, the growth trend model [coefficient of determination (R2) = 0.9637] suggests that there is a significant relationship between the year and the number of publications. The model predicts that 503 publications will be published in 2023.

### 3.2. Analysis of publication trends

**Figure 3.**
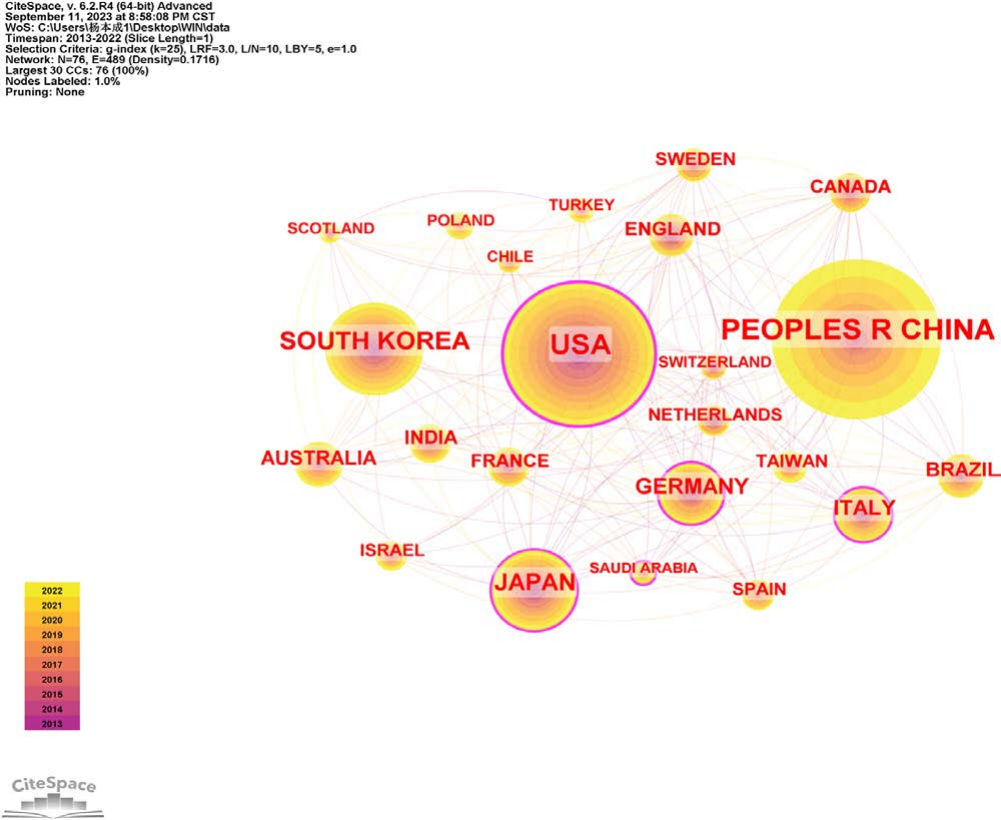
Country/region collaboration network of research on osteoimmunology and osteoporosis.

Between 2013 and 2022, 76 countries published articles in this field. The network diagram of cooperation between countries consists of 76 nodes and 489 link lines (Fig. 3). The larger the node is, the greater the number of published papers. When the centrality value is greater than 0.1, a purple circle is placed around the node. Table 1 lists the 10 countries with the largest number of publications in this field over the past decade. China ranked first, with 1072 publications, accounting for 33.31% of the total number of publications. The United States published 827 articles, accounting for 25.30% of the total, ranking second. South Korea ranked third, with 374 papers, accounting for 11.62% of the total papers. These were followed by Japan (287, 8.92%) and Germany (162, 5.03%). The greater the centrality is, the closer the cooperation between countries. The top 5 countries in this regard were the United States (0.37), Italy (0.16), Saudi Arabia (0.13), Germany (0.12), and Japan (0.11).

**Table 1:** Countries/regions, institutions, and author ranked by publications and centrality.

Item	Rank	Name	Publications	Name	Centrality
Countries/region	1	China	1072 (33.31%)	USA	0.37
	2	USA	827 (25.30%)	Italy	0.16
	3	South Korea	374 (11.62%)	Saudi Arabia	0.13
	4	Japan	287 (8.92%)	Germany	0.12
	5	Germany	162 (5.03%)	Japan	0.11
	6	Italy	132 (4.10%)	Egypt	0.11
	7	Australia	101 (3.13%)	China	0.09
	8	Brazil	101 (3.13%)	Australia	0.09
	9	Enghland	89 (2.76%)	Enghland	0.07
	10	India	77 (2.39%)	South Korea	0.06
Institutions	1	Shanghai Jiaotong University	85 (2.64%)	University of California System	0.18
	2	Harvard University	76 (2.36%)	University of Pennsylvania	0.18
	3	University of California System	71 (2.21%)	National Institutes of Health(NIH)	0.16
	4	Veterans Health Administration	68 (2.11%)	Forsyth Institute	0.11
	5	US Department of Veterans Affairs	68 (2.11%)	University of Texas System	0.1
	6	UDICE-French Research Universities	56 (1.74%)	Seoul National University	0.08
	7	Soochow University-China	56 (1.74%)	Centre National de la Recherche Scientifique	0.08
	8	Sichuan University	54 (1.68%)	University of Erlangen Nuremberg	0.08
	9	Chinese Academy of Sciences	52 (1.62%)	Vrije Universiteit Amsterdam	0.08
	10	Universidade de Sao Paulo	50 (1.55%)	Aarhus University	0.08
Authors	1	Xu Jiake	22 (0.07%)	Xu Jiake	0
	2	Oh Jaemin	18 (0.06%)	Oh Jaemin	0
	3	Yang Hulin	17 (0.05%)	Yang Hulin	0
	4	Kim Ju-Young	17 (0.05%)	Kim Ju-Young	0
	5	Hajishengallis George	17 (0.05%)	Hajishengallis George	0

**Figure 4.**
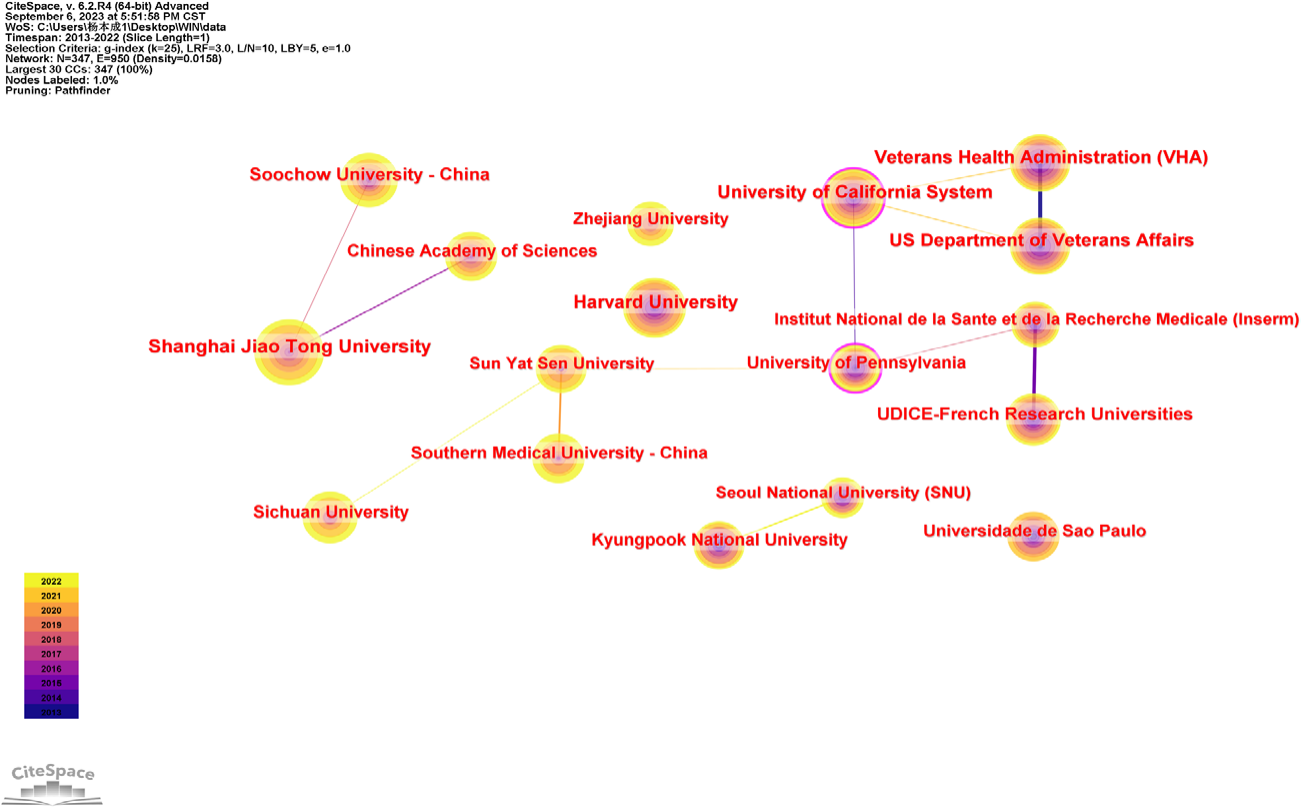
Institutions’ collaboration network of research on osteoimmunology and osteoporosis.

During the study period, a total of 347 institutions were involved in the publishing of papers in osteoimmunology and OP. Figure 4 shows the cooperation network diagram between the main institutions, covering 347 nodes and 950 link lines. Table 1 lists the top 10 institutions with the number of published documents. The institutions with more than 60 publications were Shanghai Jiaotong University (85, 2.64%), Harvard University (76, 2.36%), the University of California System (71, 2.21%), Veterans Health Administration (68, 2.11%), and US Department of Veterans Affairs (68, 2.11%). The larger the centrality value is, the stronger the cooperation between institutions. The top 5 institutions in this regard are the University of California System (0.18), University of Pennsylvania (0.18), National Institutes of Health (NIH) (0.16), Forsyth Institute (0.11), and University of Texas System (0.1).

**Figure 5.**
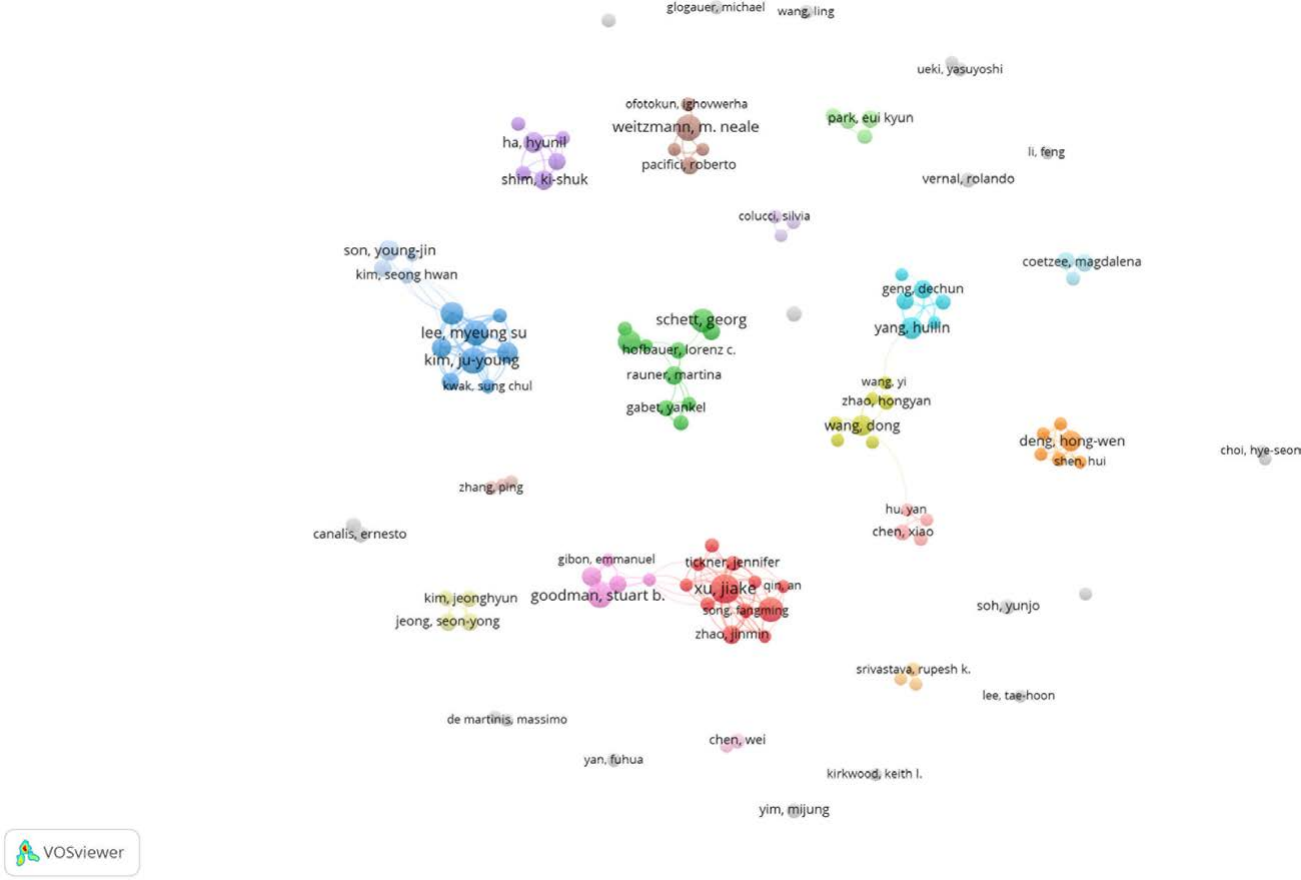
Author collaboration network of osteoimmunology and osteoporosis research.

From 2013 to 2022, 502 authors published papers in the field of osteoimmunology and OP. Figure 5 shows the collaboration network diagram between the main authors. Table 1 lists the top 5 authors: Xu, Jiake (22, 0.07%); Oh, Jaemin (18, 0.06%); Yang, Hulin (17, 0.05%); Kim, Ju-Young (17, 0.05%); and Hajishengallis, George (17, 0.05%).

## 4. Research topic analysis

### 4.1. Analysis of highly cocited references

**Figure 6.**
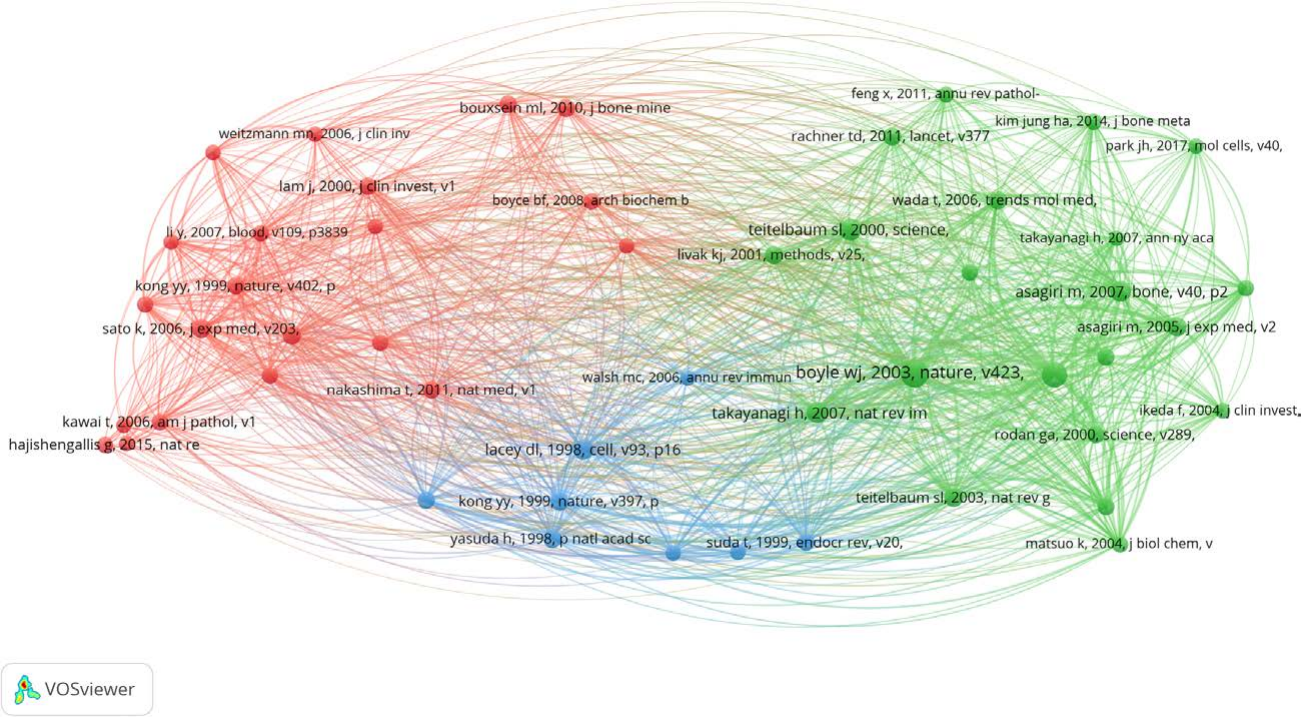
Visualization of a clustering map of cocited references.

Highly cited references refer to the references that are cited by researchers at a high frequency. Through the analysis of highly cocited references by Vosviewer drawing, we can understand the common research basis in the field of osteoimmunology and OP. According to Vosviewer, a total of 114,068 papers were cited by the papers included in this study, and when the number of citations was set to 65, there were still 51 articles. The high-cocitation reference network diagram is divided into 3 clusters, corresponding to the 3 colors in the figure (Fig. 6). The red cluster is mainly about basic experimental research on OC formation, including Th17 cells^[42]^ and the RANKL/RANK/OPG signaling pathway.^[43,44]^ The green cluster is mainly about reviews of OC differentiation and activation. The blue cluster is mainly the regulation of osteoprotegerin ligands on OC formation.^[45,46]^

Table 2 lists the top 10 articles with the highest number of citations in the cocited references. The main direction of basic experimental research is the molecular mechanism of osteoimmunology and OP, such as RANKL/RANK/OPG, Th17, and NFATc1. Among them, “Osteoclast differentiation and activation” published in Nature, is the most frequently cocited article. William J Boyle et al proposed insights into how the RANKL signaling pathway induces osteoclast formation and how hormone signals lead to changes in bone structure.^[16]^ D L Lacey’s article points out that osteoprotegerin ligands can stimulate osteoclast precursor cells in the bone marrow to form osteoclasts under the regulation of CSF-1, and OPG can block this process and inhibit the formation of OCs and related bone resorption.^[46]^ Kojiro Sato et al found that T17 cells can produce a large amount of IL-17 and promote the formation of OCs by inducing RANKL on osteoblasts to bind to RANK in a study of the effects of various Th cell subsets on bone destruction.^[42]^ Masataka Asagiri et al suggested that NFTAc1 is a transcription factor that is crucial for T-cell differentiation and osteoclast differentiation and that osteoclast precursor cells lacking NFTAc1 cannot differentiate into OCs.^[47]^

**Table 2:** Top 10 highly cocited references.

ltem	Ranking	Title	Citations	Year
Co-references	1	Osteoclast differentiation and activation	521	2003
	2	Induction and activation of the transcription factor NFATc1 (NFAT2) integrate RANKL signaling in terminal differentiation of osteoclasts	354	2002
	3	Bone resorption by osteoclasts	220	2000
	4	Osteoimmunology: shared mechanisms and crosstalk between the immune and bone systems	200	2007
	5	The molecular understanding of osteoclast differentiation	182	2007
	6	Osteoprotegerin ligand is a cytokine that regulates osteoclast differentiation and activation	170	1998
	7	Th17 functions as an osteoclastogenic helper T cell subset that links T cell activation and bone destruction	150	2006
	8	Autoamplification of NFATc1 expression determines its essential role in bone homeostasis	136	2005
	9	Genetic regulation of osteoclast development and function	135	2003
	10	Osteoclast differentiation factor is a ligand for osteoprotegerin osteoclastogenesis-inhibitory factor and is identical to TRANCE/RANKL	127	1998

### 4.2. Analysis of highly cited references

Table 3 shows the top 10 highly cited references related to osteoimmunology and OP. According to this table, Walsh, Matthew C’s article named “Biology of the RANKL–RANK–OPG system in immunity, bone, and beyond” was cited 415 times and was the most cited paper.^[27]^ This publication reviews the key regulatory functions of RANKL–RANK–OPG in organogenesis, bone homeostasis regulation, immunity and osteoimmunology, and clarifies the key regulatory relationship between bone and immunity, which is the most important molecular pathway in osteoimmunology and OP. Although the role of certain cytokines has been identified, such as IL-6, IL-17, IL-23, TNF-a, etc,^[48]^ RANKL is still the most critical pathway between bone immune network cells. Jau-Yi Li’s paper named “Sex steroid deficiency-associated bone loss is microbiota dependent and regenerating by probiotics” was cited 327 times, ranking second.^[49]^ This article proposes that sex steroid deficiency causes increased intestinal permeability, leading to the expansion of Th17 cells and upregulation of osteoclast-inducing cytokines in the small intestine and bone marrow (TNF-α, RANKL, IL-17, etc). Probiotics can reduce intestinal permeability and inhibit the production of cytokines in the intestine and bone marrow and bone loss caused by sex steroid deficiency.^[49]^ In addition, reactive oxygen species (ROS), WNT, LGR4, exosomes, etc, are also current research hotspots.

**Table 3:** Top 10 highly cited references.

ltem	Ranking	Title	Citations	Year
High-cited references	1	Biology of the RANKL–RANK–OPG system in immunity, bone, and beyond	415	2014
	2	Sex steroid deficiency-associated bone loss is microbiota dependent and prevented by probiotics	327	2016
	3	Reactive oxygen species and oxidative stress in osteoclastogenesis, skeletal aging and bone diseases	252	2015
	4	WNT1 mutations in early-onset osteoporosis and osteogenesis imperfecta	246	2013
	5	LGR4 is a receptor for RANKL and negatively regulates osteoclast differentiation and bone resorption	226	2016
	6	Recent advances in osteoclast biology	224	2018
	7	The microbial metabolite butyrate stimulates bone formation via T regulatory cell-mediated regulation of WNT10B expression	207	2018
	8	Immunology of osteoporosis: A mini-review	185	2016
	9	Wnt4 signaling prevents skeletal aging and inflammation by inhibiting nuclear factor-kappa B	163	2014
	10	Reversal of osteoporotic activity by endothelial cell-secreted bone targeting and biocompatible exosomes	159	2019

### 4.3. Analysis of co-occurring keywords and burst terms

**Figure 7.**
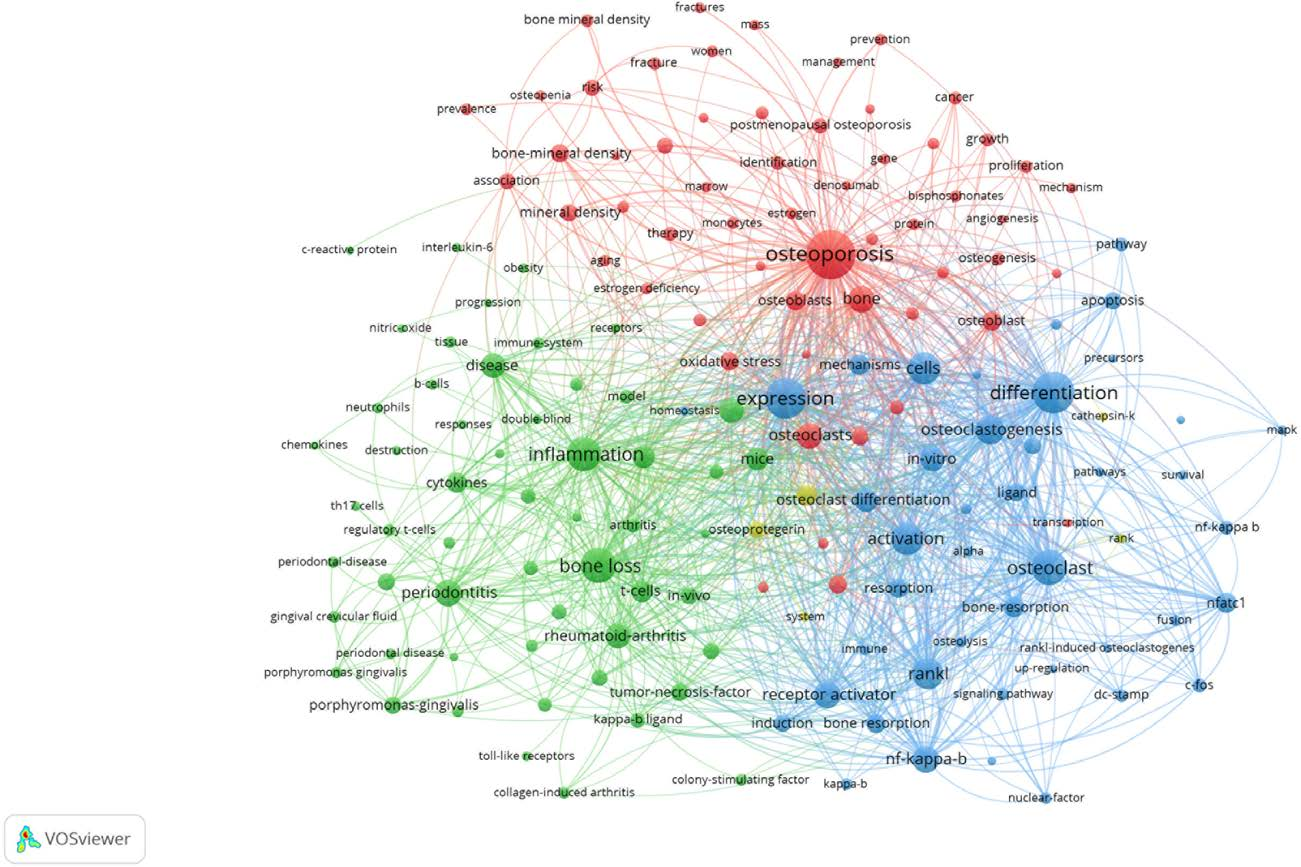
Keyword co-occurrence map of osteoimmunology and osteoporosis.

**Figure 8.**
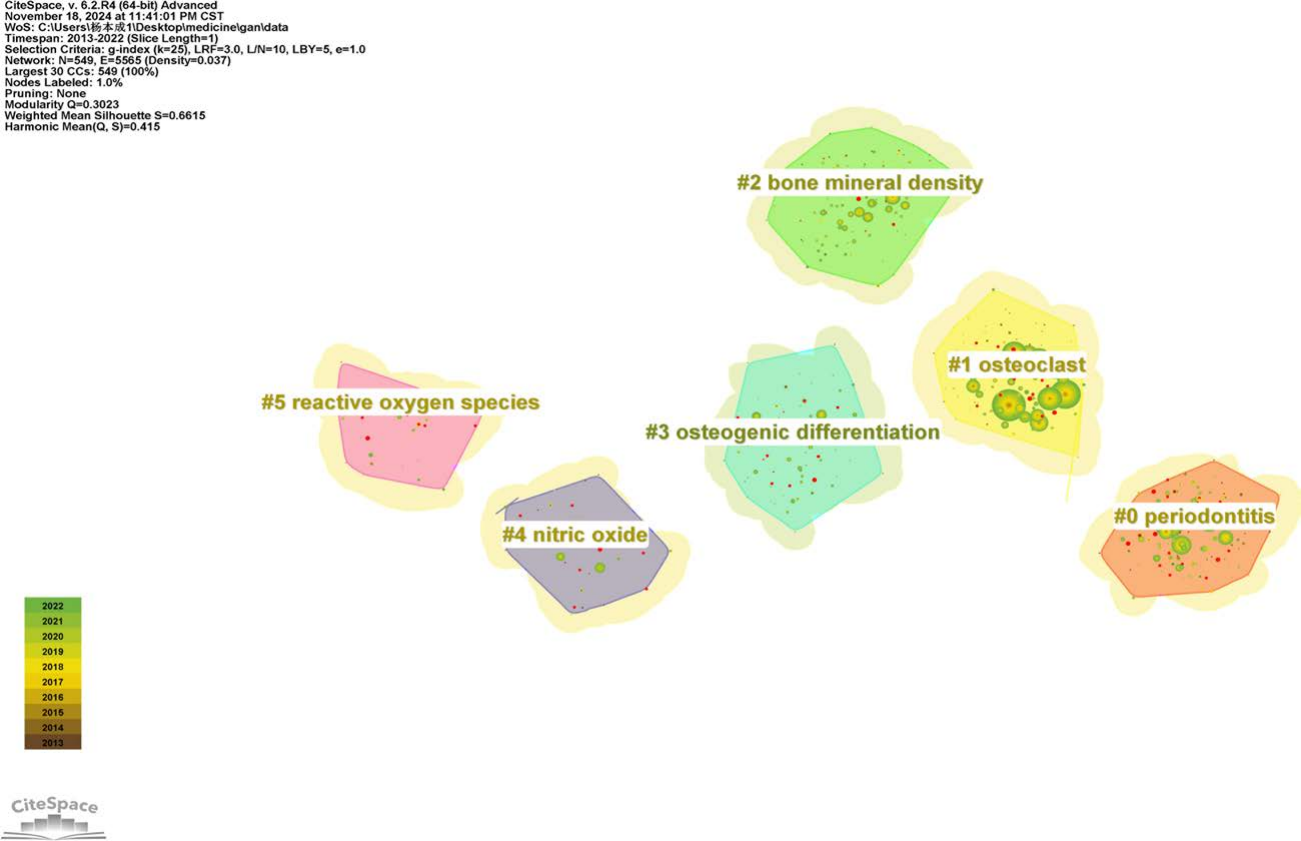
Keyword cluster map of osteoimmunology and osteoporosis.

**Figure 9.**
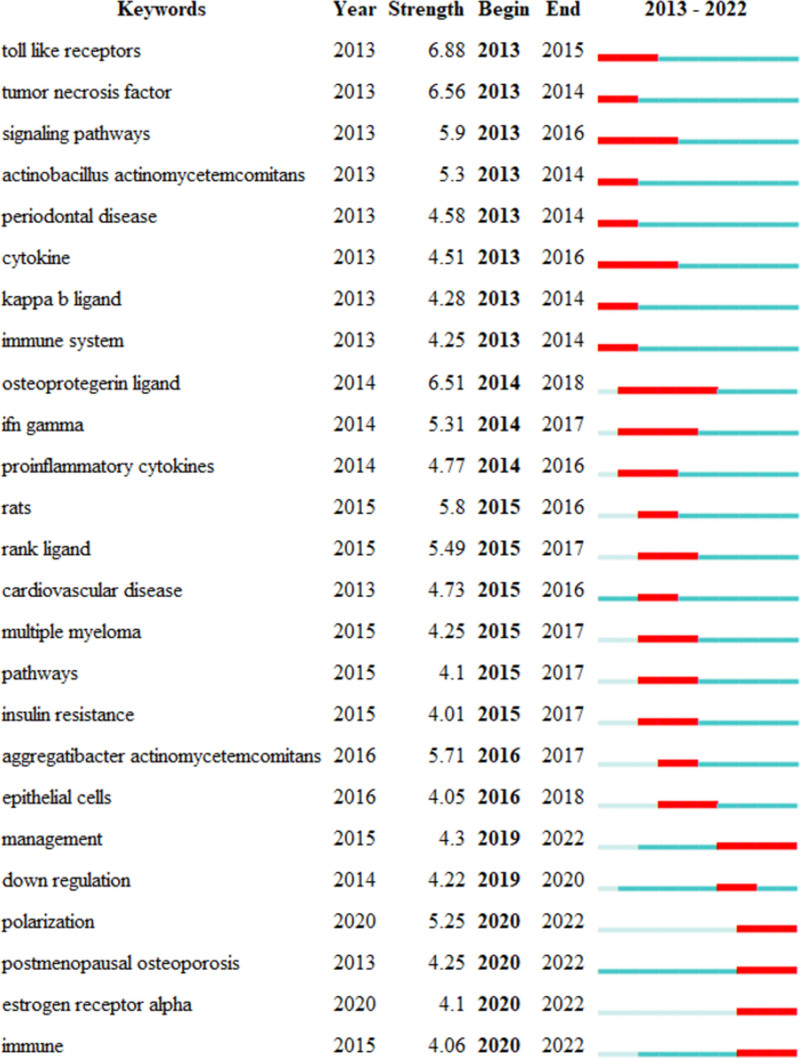
Top 25 keywords with the strongest citation bursts.

We analyzed keywords related to osteoimmunology and osteoporosis from 2013 to 2022. As shown in Figure 7, the research hotspots in this field can be understood through keyword co-occurrence analysis. Table 4 shows the top 20 keywords by frequency and centrality. Among them, 6 terms had frequencies over 400, including “expression,” “differentiation,” “bone loss,” “osteoporosis,” “cells,” and “activation.” According to the centrality ranking, the top 5 keywords include “activation,” “osteoblasts,” “growth,” “cfos,” and “estrogen deficiency.” The greater the centrality is, the stronger the correlation between the items in the 2 fields. We can use keyword clustering analysis to classify and summarize the research topics. We used CiteSpace to cluster terms and obtained 6 clusters. As shown in Figure 8 and Table 5, they are “#0 periodontitis,” “#1 osteoclast,” “#2 bone mineral density,” “#3 osteogenic differentiation,” “#4 nitric oxide,” and “#5 reactive oxygen species.” When silhouette is greater than 0.5, the clustering is reasonable. When silhouette is greater than 0.7, the cluster is persuasive and efficient.^[41]^ Keyword bursts can predict the development trend of a certain field, indicating a sudden increase in research content over a period of time. Figure 9 shows the 25 keywords with the largest burst intensity. The red line indicates the duration of the keyword burst. As shown in the figure, the keyword theme gradually changed from “tumor necrosis factor,” “toll like receptors,” and “signaling pathways” to “postmenopausal osteoporosis,” “estrogen receptor alpha,” and “immune.” These all reflect the latest research trends.

**Table 4:** The top 20 keywords in terms of frequency and centrality.

Ranking	Keyword	Frequency	Keyword	Centrality
1	Expression	658	Bone marrow	0.04
2	Differentiation	657	Osteoblasts	0.03
3	Bone loss	481	Growth	0.03
4	Osteoporosis	459	CFOS	0.03
5	Cells	419	Estrogen deficiency	0.03
6	Activation	405	Cancer	0.03
7	Rheumatoid arthritis	321	Women	0.03
8	Receptor activator	301	Gingival crevicular fluid	0.03
9	NF Kappa B	300	Colony stimulating factor	0.03
10	Bone resorption	298	B cells	0.03
11	In vitro	241	Collagen induced arthritis	0.03
12	Disease	237	Marrow	0.03
13	Inflammation	231	Vitamin D	0.03
14	Osteoclast differentiation	222	Breast cancer	0.03
15	T cells	207	Alpha	0.03
16	Bone mineral density	202	Homeostasis	0.03
17	RANKL	197	Disease	0.02
18	Mice	195	T cells	0.02
19	Bone	182	Porphyromonas gingivalis	0.02
20	Macrophages	177	Tumor necrosis factor	0.02

**Table 5:** Keyword cluster analysis.

Cluster	Size	Silhouette	Mean year	Label (LLR)	Other keywords
#0	151	0.669	2015	Periodontitis	Porphyromonas gingivalis; innate immunity; oral bone loss; bone mineral density; experimental autolmmue myasthena gravis
#1	129	0.64	2015	Osteoclast	osteoclast; rankl; nfatcl; bone loss; miconazole
#2	118	0.588	2015	Bone mineral density	Bone mineral density; lysosomal membrane permeabilization; plant-produced monoclonal antibody; nicotiana benthamiana; alpha V integrin
#3	72	0.662	2017	Osteogenic differentiation	Bone remodeling; cell differentiation; gene therapy; arimal models; ceramic scaffolds
#4	44	0.796	2015	Nitric oxide	Bone marrow; macrophages; T cells; dendritic cells; monocytes
#5	35	0.783	2016	Reactive oxygen species	Reactive oxygen species; receptor activator; nuclear factor-kappa B; advanced glycation end products; one resorption

### 4.4. Analysis of high-yield journals

**Figure 10.**
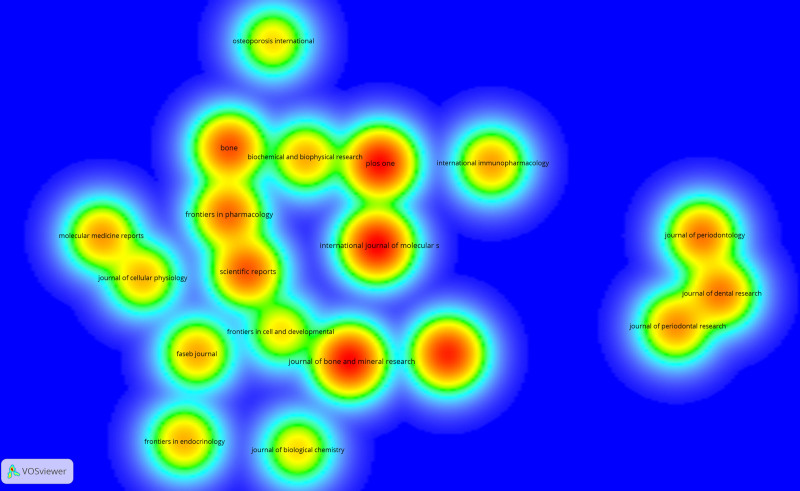
Journal density map.

Table 6 lists the top 10 journals in the field of osteoimmunology and OP. Journals with more than 60 papers published in this field include International Journal of Molecular Sciences (89), Journal of Bone and Mineral Research (87), PLoS One (86), Frontiers in Immunology (76), and Bone (61). Journal of Bone and Mineral Research (3791) and Frontiers in Immunology (2430) were cited more than 2000 times. Figure 10 shows 19 journals that published more than 25 papers on this topic in 821 journals worldwide over the past decade.

**Table 6:** Top 10 high-yield journals.

Ranking	Journal	Publications	Citaions
1	International Journal of Molecular Sciences	89	1284
2	Journal of Bone and Mineral Research	87	3791
3	Plos one	86	1692
4	Frontiers in Immunology	76	2430
5	Bone	61	1893
6	Scientific Reports	59	1274
7	Frontiers in Pharmacology	53	480
8	Journal of Periodontology	49	945
9	Journal of Dental Research	48	1385
10	Journal of Periodontal Research	42	954

## 5. Discussion

### 5.1. Analysis of publication trends and partnership

Over the past decade, there has been a steady increase in the number of articles on osteoimmunology and OP each year, indicating that the topic remains a research hotspot. China had more than 1000 publications, is the country with the largest number of publications and has made great contributions to the research in this field. China is followed by the United States and South Korea, whose number of publications related to this topic were 827 and 374, respectively.

Through bibliometric analysis, we can understand the state of cooperation among authors, institutions, and countries. The centrality value represents the intensity of cooperation. The greater the centrality display, the closer the cooperation is. In terms of mutual cooperation among countries, the United States, Italy, Saudi Arabia, Germany, and Japan are the top 5 countries in the central ranking, indicating that they have close cooperation with other countries. While China has the largest number of publications, its collaboration with other countries lags far behind. The United States has the greatest centrality and cooperates most closely with other countries. In terms of institutional cooperation, the University of California System, University of Pennsylvania, National Institutes of Health (NIH), Forsyth Institute, and University of Texas System are the top 5 institutions. Interestingly, they are all U.S. research institutions. The United States, as the most developed country in the world, has the most advanced basic experimental facilities and technical conditions, and it also has the most top scientific researchers and sufficient scientific research funds. In addition, this may be related to the country’s emphasis on this field and financial investment. Therefore, the United States occupies a leading position in this research field. In the future, with the sustained economic growth of countries such as Europe and Asia and the increasing emphasis on the medical and health field, especially China, as the country with the largest number of publications in this field. The gap between the United States and other countries will gradually decrease. Through the analysis of the collaboration between authors, it is found that the centrality of all authors is 0. This indicates that there is a lack of academic exchanges and cooperation among researchers in this field. This is related to the degree of interest of researchers in this field and the unique views of scholars in different fields in their respective fields. In addition, this may also be related to the investment of state and scientific research institutions in this field. Strengthening cooperation among researchers can promote exchanges between disciplines and derive new research directions. Top scientists can help other researchers discover new directions and guidelines. Therefore, the cooperation between researchers needs to be strengthened. Enhancing the cooperation between authors may promote the progress of the study.

### 5.2. Research hot spot and trend

Through bibliometric analysis, we can understand the research hotspots and trends in this field. By analyzing highly cocited references and highly cited references, we found that the research focus of osteoimmunology and OP is mainly about the molecular mechanism of OC activation and differentiation, including RANKL/RANK/OPG, WNT, Th17, NFATc1, ROS, etc. Through the keyword burst, we found that “postmenopausal osteoporosis,” “estrogen receptor alpha,” and “immune” are the current research hotspots and trends. Through keyword clustering analysis, we found that researchers are more concerned with the mechanism and role of inflammatory cytokines in the fields of osteoimmunology and osteoporosis. This is not only the current research hotspot but also the future research direction and trend. At the same time, probiotics and intestinal flora should also be concerned. Previous studies have confirmed that intestinal flora can regulate the body’s immune homeostasis and affect bone metabolism, which is closely related to this field. In addition, researchers have found that Chinese medicinal plants can regulate the immune mechanism of the human body and affect the occurrence and progression of osteoporosis. At present, researchers have gradually deepened their research on osteoimmunology and osteoporosis. It is not limited to the study of a single disease but connects diseases with common mechanisms. In the past 2 years, scholars have gradually become interested in postmenopausal osteoporosis. Moreover, researchers can grasp the research progress of osteoimmunology and OP through the most commonly used terms in the past decade.

### 5.3. RANKL/RANK/OPG

The root cause of OP is abnormal OC differentiation.^[50]^ Little is known about the exact differentiation pathway and molecular pathway of OCs. Current studies have shown that RANKL/RANK/OPG plays an indispensable role in OC differentiation.^[51]^ The discovery of RANKL/RANK/OPG undoubtedly promoted the development of osteoimmunology and closely connected osteoimmunology with OP.^[52]^ The key stage of OC differentiation is the binding of RANKL to RANK on osteoclast precursor cells and the activation of the bridging protein tumor necrosis factor receptor associated factor (TRAF6) in the RANK cytoplasmic region; then, TRAF6 further activates and initiates downstream signaling.^[8]^ Subsequently, NF-κB, MAPK, PI3K/AKT, Ca^2+^, and ROS signals are also activated.^[16,19,53]^ In this pathway, receptor activator of nuclear factor-κB ligand (RANKL) and macrophage colony stimulating factor (M-CSF) are key regulatory molecules.^[54]^ However, studies have shown that the differentiation and formation of macrophages and OCs are impaired after mutation of the M-CSF gene, which confirms that bone cells and immune cells are of the same origin.^[55]^ Recent studies have shown that bone cells may be the most important source of RANKL.^[44,56]^ OPG, which belongs to the tumor necrosis factor receptor superfamily, is a glycoprotein that regulates bone resorption. It is produced by osteoblasts, dendritic cells, B cells, etc., and can compete with RANK to bind RANKL and inhibit bone resorption.^[46,57]^ At present, long-term use of anti-osteoporosis drugs can cause some adverse reactions.^[8]^ Targeted treatment of OP through the RANKL/RANK/OPG pathway and downstream cascade transduction signaling pathway is a hot spot of current research. As an anti-RANKL antibody, denosumab has been used in clinical practice. These pathways may provide new ideas for creating new targets for the treatment of OP. It is believed that more targeted drugs will be developed in the future, and more patients will benefit from them.

### 5.4. Gut flora and probiotics

In 2012, Klara Sjögren et al found that gut microbiota played an important role in the regulation of bone mass.^[58]^ Studies have shown that there are significant differences in intestinal microbes between patients with inflammatory bowel disease and healthy people.^[59]^ Recently, experiments have shown that sex steroid deficiency can lead to increased intestinal permeability and upregulate the levels of TNF, RANKL and IL-17 in the intestine and bone marrow, causing bone loss, and inflammatory pathways are necessary in this process.^[49]^ The experiment also found that probiotics can promote intestinal epithelial tightening, induce the expression of related proteins, reduce intestinal permeability, and prevent bone loss.^[49]^ However, in a clinical study, researchers treated 50 postmenopausal women with placebo or probiotic intervention for 6 months and found no significant change in bone mineral density in all cases. However, TNF levels and the bone turnover index decreased in the probiotic group.^[60]^ This result may be due to the intervention time being too short. Previous studies have also demonstrated that probiotics improve intestinal barrier function and modulate the host immune response.^[61,62]^ Hsu, Emory et al pointed out that probiotics can increase the expression level of tight junction proteins, thereby reducing antigen transmission and immune cell activation.^[63]^ Currently, most studies on gut microbiota and probiotics in this field are still in the animal experimental stage.^[63]^ Probiotics, as low-cost and high-yield drugs, may become a new way to treat OP.

## 6. Limitations

There are still some limitations in this study. First, we only searched for literature in the Web of Science Core Collection database, which will result in missing some articles. However, as the most common database, Web of Science Core Collection has a strict screening mechanism, only includes important academic journals in various disciplines, and it has a strong citation analysis function and citation network, with sufficient data to explain the relationship between osteoimmunology and osteoporosis. Second, we only selected English publications and excluded papers in other languages, which results in some bias. Third, we cannot ensure that every retrieved article met the search requirements.

## 7. Conclusion

This study used bibliometric and visual analysis to analyze 3218 articles related to bone immunology and OP published in the Web of Science Core Collection database from 2013 to 2022. The number of articles in the field continues to rise steadily each year, indicating that osteoimmunology and OP are still hot fields. Among them, China is the country with the most publications. Shanghai Jiaotong University is the institution with the largest publication volume, and Xu, Jiake is the most prolific author in the examined papers. The United States cooperates most closely with other countries and institutions, which comes from its strong scientific and technological strength and economic strength. Through the analysis of literature and keywords, we found that the RANKL/RANK/OPG pathway is still the focus of current research, and some drugs have been developed on the basis of this pathway in clinical practice. Researchers should pay more attention to the RANKL/RANK/OPG pathway in future research. Blocking the important cytokines in the signaling pathway and inhibiting certain inflammatory cytokines can treat or delay the occurrence of osteoporosis, which has great development potential. However, this requires a large number of animal experiments and clinical trials to further verify. In addition, intestinal flora and Chinese medicinal plants also play an important role in the field of osteoimmunology and osteoporosis, which should be paid attention to. The study provides a comprehensive summary of articles related to this topic over the past decade, pointing out the current research focus and direction. This study can provide a reference for researchers in this field.

## Acknowledgments

Thanks for the fund support provided by Bethune Shiyao Osteoporosis Research Fund Project.

## Author contributions

**Conceptualization:** Bencheng Yang, Fusheng Xiong.

**Data curation:** Bencheng Yang, Fusheng Xiong.

**Formal analysis:** Fusheng Xiong.

**Funding acquisition:** Fusheng Xiong.

**Investigation:** Fusheng Xiong.

**Methodology:** Fusheng Xiong.

**Project administration:** Fusheng Xiong.

**Resources:** Bencheng Yang, Fusheng Xiong.

**Software:** Bencheng Yang, Mingshuai Tan, Fusheng Xiong.

**Supervision:** Mingshuai Tan, Fusheng Xiong.

**Validation:** Mingshuai Tan.

**Visualization:** Bencheng Yang.

**Writing – original draft:** Bencheng Yang, Mingshuai Tan.

**Writing – review & editing:** Bencheng Yang, Mingshuai Tan.

## References

[1] Bijlsma AY Meskers CGM Westendorp RGJ Maier AB. Chronology of age-related disease definitions: osteoporosis and sarcopenia. Ageing Res Rev. 2012;11:320–4. 22306229 10.1016/j.arr.2012.01.001

[2] Bogunovic L Cherney SM Rothermich MA Gardner MJ. Biomechanical considerations for surgical stabilization of osteoporotic fractures. Orthop Clin North Am. 2013;44:183–200. 23544823 10.1016/j.ocl.2013.01.006

[3] Yaacobi E Sanchez D Maniar H Horwitz DS. Surgical treatment of osteoporotic fractures: an update on the principles of management. Injury. 2017;48:S34–40. 10.1016/j.injury.2017.08.036 28882375

[4] Wright NC Looker AC Saag KG. The recent prevalence of osteoporosis and low bone mass in the United States based on bone mineral density at the femoral neck or lumbar spine. J Bone Miner Res. 2014;29:2520–6. 24771492 10.1002/jbmr.2269 PMC4757905

[5] Yu B Wang C-Y. Osteoporosis: the result of an ‘aged’ bone microenvironment. Trends Mol Med. 2016;22:641–4. 27354328 10.1016/j.molmed.2016.06.002 PMC4969144

[6] Anthamatten A Parish A. Clinical update on osteoporosis. J Midwifery Womens Health. 2019;64:265–75. 30869832 10.1111/jmwh.12954

[7] Cosman F de Beur SJ LeBoff MS. Clinician’s guide to prevention and treatment of osteoporosis. Osteoporos Int. 2014;25:2359–81. 25182228 10.1007/s00198-014-2794-2 PMC4176573

[8] Qu Z Zhang B Kong L. Receptor activator of nuclear factor-κB ligand-mediated osteoclastogenesis signaling pathway and related therapeutic natural compounds. Front Pharmacol. 2022;13:1043975. 36438811 10.3389/fphar.2022.1043975 PMC9683337

[9] Rachner TD Khosla S Hofbauer LC. Osteoporosis: now and the future. Lancet. 2011;377:1276–87. 21450337 10.1016/S0140-6736(10)62349-5 PMC3555696

[10] Clarke BL Khosla S. Physiology of bone loss. Radiol Clin North Am. 2010;48:483–95. 20609887 10.1016/j.rcl.2010.02.014 PMC2901252

[11] Manolagas SC. From estrogen-centric to aging and oxidative stress: a revised perspective of the pathogenesis of osteoporosis. Endocr Rev. 2010;31:266–300. 20051526 10.1210/er.2009-0024 PMC3365845

[12] Ding K Hua F Ding W. Gut microbiome and osteoporosis. Aging Dis. 2020;11:438–47. 32257552 10.14336/AD.2019.0523 PMC7069453

[13] Behera J Ison J Tyagi SC Tyagi N. The role of gut microbiota in bone homeostasis. Bone. 2020;135:115317. 32169602 10.1016/j.bone.2020.115317 PMC8457311

[14] Pacifici R. Bone remodeling and the microbiome. Cold Spring Harb Perspect Med. 2018;8:a031203. 28847904 10.1101/cshperspect.a031203 PMC5880157

[15] Rodan GA Martin TJ. Therapeutic approaches to bone diseases. Science. 2000;289:1508–14. 10968781 10.1126/science.289.5484.1508

[16] Boyle WJ Simonet WS Lacey DL. Osteoclast differentiation and activation. Nature. 2003;423:337–42. 12748652 10.1038/nature01658

[17] Asagiri M Takayanagi H. The molecular understanding of osteoclast differentiation. Bone. 2007;40:251–64. 17098490 10.1016/j.bone.2006.09.023

[18] McDonald MM Khoo WH Ng PY. Osteoclasts recycle via osteomorphs during RANKL-stimulated bone resorption. Cell. 2021;184:1330–47.e13. 33636130 10.1016/j.cell.2021.02.002 PMC7938889

[19] Xu F Teitelbaum SL. Osteoclasts: new insights. Bone Res. 2013;1:11–26. 26273491 10.4248/BR201301003 PMC4472093

[20] Takayanagi H. Osteoimmunology and the effects of the immune system on bone. Nat Rev Rheumatol. 2009;5:667–76. 19884898 10.1038/nrrheum.2009.217

[21] Limmer A Wirtz D. Osteoimmunology: influence of the immune system on bone regeneration and consumption. Z Orthop Unfall. 2017;155:273–80. 28683495 10.1055/s-0043-100100

[22] Weitzmann MN Ofotokun I. Physiological and pathophysiological bone turnover – role of the immune system. Nat Rev Endocrinol. 2016;12:518–32. 27312863 10.1038/nrendo.2016.91 PMC5857945

[23] Bozec A Zaiss MM. T regulatory cells in bone remodelling. Curr Osteoporos Rep. 2017;15:121–5. 28432597 10.1007/s11914-017-0356-1

[24] Zhao L Huang L Zhang X. Osteoimmunology: memorandum for rheumatologists. Sci China Life Sci. 2016;59:1241–58. 27650950 10.1007/s11427-016-5105-7

[25] Guerrini MM Takayanagi H. The immune system, bone and RANKL. Arch Biochem Biophys. 2014;561:118–23. 24929185 10.1016/j.abb.2014.06.003

[26] Okamoto K Takayanagi H. Osteoimmunology. Cold Spring Harb Perspect Med. 2019;9:a031245. 29610150 10.1101/cshperspect.a031245 PMC6314075

[27] Walsh MC Choi Y. Biology of the RANKL-RANK-OPG system in immunity, bone, and beyond. Front Immunol. 2014;5:511. 25368616 10.3389/fimmu.2014.00511 PMC4202272

[28] Zhang W Dang K Huai Y Qian A. Osteoimmunology: the regulatory roles of T lymphocytes in osteoporosis. Front Endocrinol. 2020;11:456. 10.3389/fendo.2020.00465 PMC7431602 32849268

[29] Fuller K Murphy C Kirstein B Fox SW Chambers TJ. TNFα potently activates osteoclasts, through a direct action independent of and strongly synergistic with RANKL. Endocrinology. 2002;143:1108–18. 11861538 10.1210/endo.143.3.8701

[30] Li Y Toraldo G Li A. B cells and T cells are critical for the preservation of bone homeostasis and attainment of peak bone mass in vivo. Blood. 2007;109:3839–48. 17202317 10.1182/blood-2006-07-037994 PMC1874582

[31] Luo CY Wang L Sun C Li DJ. Estrogen enhances the functions of CD4+CD25+Foxp3+ regulatory T cells that suppress osteoclast differentiation and bone resorption in vitro. Cell Mol Immunol. 2010;8:50–8. 21200384 10.1038/cmi.2010.54 PMC4002989

[32] Dar HY Shukla P Mishra PK. *Lactobacillus acidophilus* inhibits bone loss and increases bone heterogeneity in osteoporotic mice via modulating Treg-Th17 cell balance. Bone Rep. 2018;8:46–56. 29955622 10.1016/j.bonr.2018.02.001 PMC6019967

[33] Yuan F-L Li X Lu W-G. Regulatory T cells as a potent target for controlling bone loss. Biochem Biophys Res Commun. 2010;402:173–6. 20920469 10.1016/j.bbrc.2010.09.120

[34] Tanaka Y. Clinical immunity in bone and joints. J Bone Miner Metab. 2018;37:2–8. 30324535 10.1007/s00774-018-0965-5

[35] Wan X Wang X Pang R. Mapping knowledge landscapes and emerging trends of the links between osteoarthritis and osteoporosis: a bibliometric analysis. Front Public Health. 2022;10:1019691. 36600941 10.3389/fpubh.2022.1019691 PMC9806179

[36] Li F Xie W Han Y Li Z Xiao J. Bibliometric and visualized analysis of exercise and osteoporosis from 2002 to 2021. Front Med (Lausanne). 2022;9:944444. 36569140 10.3389/fmed.2022.944444 PMC9773261

[37] Jiang B Feng C Li C Tu C Li Z. A bibliometric and visualization analysis of glucocorticoid-induced osteoporosis research from 2012 to 2021. Front Endocrinol. 2022;13:961471. 10.3389/fendo.2022.961471 PMC9388768 35992120

[38] Ma C Su H Li H. Global research trends on prostate diseases and erectile dysfunction: a bibliometric and visualized study. Front Oncol. 2021;10:627891. 33643922 10.3389/fonc.2020.627891 PMC7908828

[39] Cobo MJ López-Herrera AG Herrera-Viedma E Herrera F. Science mapping software tools: review, analysis, and cooperative study among tools. J Am Soc Inf Sci Technol. 2011;62:1382–402.

[40] Ma L Ma J Teng M Li Y. Visual analysis of colorectal cancer immunotherapy: a bibliometric analysis from 2012 to 2021. Front Immunol. 2022;13:843106. 35432385 10.3389/fimmu.2022.843106 PMC9009266

[41] Zou X Sun Y. Bibliometrics analysis of the research status and trends of the association between depression and insulin from 2010 to 2020. Front Psychiatry. 2021;12:683474. 34366917 10.3389/fpsyt.2021.683474 PMC8339804

[42] Sato K Suematsu A Okamoto K. Th17 functions as an osteoclastogenic helper T cell subset that links T cell activation and bone destruction. J Exp Med. 2006;203:2673–82. 17088434 10.1084/jem.20061775 PMC2118166

[43] Lam J Takeshita S Barker JE Kanagawa O Ross FP Teitelbaum SL. TNF-α induces osteoclastogenesis by direct stimulation of macrophages exposed to permissive levels of RANK ligand. J Clin Investig. 2000;106:1481–8. 11120755 10.1172/JCI11176 PMC387259

[44] Nakashima T Hayashi M Fukunaga T. Evidence for osteocyte regulation of bone homeostasis through RANKL expression. Nat Med. 2011;17:1231–4. 21909105 10.1038/nm.2452

[45] Kong YY Yoshida H Sarosi I. OPGL is a key regulator of osteoclastogenesis, lymphocyte development and lymph-node organogenesis. Nature. 1999;397:315–23. 9950424 10.1038/16852

[46] Lacey DL Timms E Tan HL. Osteoprotegerin ligand is a cytokine that regulates osteoclast differentiation and activation. Cell. 1998;93:165–76. 9568710 10.1016/s0092-8674(00)81569-x

[47] Asagiri M Sato K Usami T. Autoamplification of NFATc1 expression determines its essential role in bone homeostasis. J Exp Med. 2005;202:1261–9. 16275763 10.1084/jem.20051150 PMC2213228

[48] Takayanagi H. New developments in osteoimmunology. Nat Rev Rheumatol. 2012;8:684–9. 23070645 10.1038/nrrheum.2012.167

[49] Li JY Chassaing B Tyagi AM. Sex steroid deficiency-associated bone loss is microbiota dependent and prevented by probiotics. J Clin Invest. 2016;126:2049–63. 27111232 10.1172/JCI86062 PMC4887186

[50] Raisz LG. Pathogenesis of osteoporosis: concepts, conflicts, and prospects. J Clin Invest. 2005;115:3318–25. 16322775 10.1172/JCI27071 PMC1297264

[51] Boyce BF Xing L. Functions of RANKL/RANK/OPG in bone modeling and remodeling. Arch Biochem Biophys. 2008;473:139–46. 18395508 10.1016/j.abb.2008.03.018 PMC2413418

[52] Takayanagi H. Osteoimmunology: shared mechanisms and crosstalk between the immune and bone systems. Nat Rev Immunol. 2007;7:292–304. 17380158 10.1038/nri2062

[53] Hu Y Li X Zhi X. RANKL from bone marrow adipose lineage cells promotes osteoclast formation and bone loss. EMBO Rep. 2021;22:e52481. 34121311 10.15252/embr.202152481 PMC8406405

[54] Ross FP Teitelbaum SL. alphavbeta3 and macrophage colony-stimulating factor: partners in osteoclast biology. Immunol Rev. 2005;208:88–105. 16313343 10.1111/j.0105-2896.2005.00331.x

[55] Yoshida H Hayashi S Kunisada T. The murine mutation osteopetrosis is in the coding region of the macrophage colony stimulating factor gene. Nature. 1990;345:442–4. 2188141 10.1038/345442a0

[56] Xiong J Onal M Jilka RL Weinstein RS Manolagas SC O’Brien CA. Matrix-embedded cells control osteoclast formation. Nat Med. 2011;17:1235–41. 21909103 10.1038/nm.2448 PMC3192296

[57] Simonet WS Lacey DL Dunstan CR. Osteoprotegerin: a novel secreted protein involved in the regulation of bone density. Cell. 1997;89:309–19. 9108485 10.1016/s0092-8674(00)80209-3

[58] Kostic AD Garrett WS. Keystone microbiome meeting 2012: a mountain top experience. EMBO Rep. 2012;13:478–80.

[59] Frank DN St Amand AL Feldman RA Boedeker EC Harpaz N Pace NR. Molecular-phylogenetic characterization of microbial community imbalances in human inflammatory bowel diseases. Proc Natl Acad Sci U S A. 2007;104:13780–5. 17699621 10.1073/pnas.0706625104 PMC1959459

[60] Jafarnejad S Djafarian K Fazeli MR Yekaninejad MS Rostamian A Keshavarz SA. Effects of a multispecies probiotic supplement on bone health in osteopenic postmenopausal women: a randomized, double-blind, controlled trial. J Am Coll Nutr. 2017;36:497–506. 28628374 10.1080/07315724.2017.1318724

[61] Anderson RC Cookson AL McNabb WC. Lactobacillus plantarum MB452 enhances the function of the intestinal barrier by increasing the expression levels of genes involved in tight junction formation. BMC Microbiol. 2010;10:316. 21143932 10.1186/1471-2180-10-316 PMC3004893

[62] Yan F Polk DB. Probiotics and immune health. Curr Opin Gastroenterol. 2011;27:496–501. 21897224 10.1097/MOG.0b013e32834baa4d PMC4006993

[63] Hsu E Pacifici R. From osteoimmunology to osteomicrobiology: how the microbiota and the immune system regulate bone. Calcif Tissue Int. 2018;102:512–21. 29018933 10.1007/s00223-017-0321-0 PMC5893441

